# 

**Published:** 1912-03-15

**Authors:** 


### ANNOUNCEMENTS.

PAPYRUS EBERS. Important Editorial Comment. Some time ago, announcement was made through the pages of this journal that Dr. Carl H. von Klein had completed an English translation of the Papyrus Ebers, and that its publication in book form depended upon an advance subscription list of one thousand names.

Dr. von Klein has spent twenty years of odd moments of a busy, useful life, mostly devoted to the literature of our profession, on this work of love, and it will be regrettable if it is now lost when so little will not only preserve it to us, the profession, but give its faithful author the satisfaction of seeing it in print.

The book will consist of 650 pages, 7x10 inches, in two colors (red and black) similar to the original, with six plates, bound in one volume. Subscriptions may be sent directly to Dr. Carl H. von Klein, Medical Department, John Crerar Library, Chicago, Illinois. Dr. von Klein will publish this volume personally, and it will be sold only on subscription.

KENTUCKY STATE DENTAL ASSOCIATION. The meeting of the Kentucky State Dental Association will be held in Louisville, May 27, 28, 29, 1912. A special attraction of talented men from out of the State will be upon the program this year and every indication points to the best meeting that has been held for many years. The Dentists of Kentucky are especially invited and a cordial invitation is extended to all ethical members of the profession.

TENNESSEE DENTAL ASSOCIATION. The forty-fifth annual meeting of the Tennessee State Dental Association will be held at Memphis, Tennessee, in the Business Men's Club rooms, June 6, 7 and 8, 1912. The Business Men's Club extend the courtesies of the club to all the visiting dentists, and the Association invite all ethical dentists to attend.

CHAS. A. TAVEL, *Pres.* J. L. MANIRE, *Sec'y.*

---

MANUFACTURER'S EXHIBIT. "The Manufacturer's Exhibit during the convention of the National Dental Association in Washington, September 10th to 13th, inclusive, will be held in the New Ebbitt Hotel, which is just across the street from the headquarters and the clinics. Specifications, plans, rates and full information can be had by addressing Dr. Clyde M. Gearhart, Chairman of the Exhibit Committee, 1616 I St., N. W., Washington, D. C."

---

TEXAS STATE DENTAL ASSOCIATION. The thirty-second annual meeting of the Texas State Dental Association will be held at Abilene, Texas, May 2, 3 and 4, 1912. Exhibitors desiring space will please address Dr. C. M. McCauley, Abilene, Texas. The clinics will be in charge of Dr. J. O. Hall, Waco, Texas, who will furnish any information relative to same. All ethical practitioners are cordially invited to attend the meeting, who will be cheerfully furnished any other information by the secretary.

H. M. DAVISON, *Pres.*,
Hubbard, Texas.
J. G. FIFE, *Sec'y*,
736 Wilson Bldg.,
Dallas, Texas.

